# Public Knowledge and Attitudes Toward Blood Donation in Georgia

**DOI:** 10.7759/cureus.86885

**Published:** 2025-06-27

**Authors:** Elisabed Chikobava, Lasha Chkhikvadze, Nika Vashakidze, Elizaveta Mikeladze, Ayesha Begum Mohamed Abdul Raheem, Keti Menabde

**Affiliations:** 1 Faculty of Medicine, American MD Program, Tbilisi State Medical University, Tbilisi, GEO

**Keywords:** blood shortage, blood transfusion, knowledge and attitudes, public health awareness, voluntary non-remunerated blood donation

## Abstract

Blood donation is vital for saving lives and supporting healthcare systems worldwide. However, many countries, including Georgia, face challenges due to insufficient blood supplies. Despite its importance, there is limited data on public knowledge and attitudes toward blood donation in Georgia. In this cross-sectional study, we surveyed 385 participants through social media to assess their knowledge, attitudes, and the barriers they face regarding blood donation. Our findings showed that only 35% of participants had good knowledge, and just 38.4% demonstrated a positive attitude. Sociodemographic factors such as gender, age, residence, education level, and medical background were significantly associated with knowledge and attitudes. The most commonly reported reasons for not donating included health issues like anemia and hypotension (49.5%), lack of opportunity (29.2%), and unwillingness to donate (12.7%). Encouragingly, 83% of participants expressed willingness to donate blood in the future. As the first study of its kind in Georgia, these results emphasize the need for educational initiatives and awareness campaigns. Improving public understanding and attitudes could help translate this willingness into higher blood donation rates, contributing to a more reliable and sufficient blood supply in the country.

## Introduction

Blood donation is vital to healthcare, supporting emergency care, surgeries, and cancer treatment, and saving millions of lives annually [1,2]. According to the World Health Organization (WHO), approximately 118.5 million units of blood are collected globally each year; however, demand continues to exceed supply [1].

Despite its importance, many countries, including Georgia, face insufficient blood donation rates [3-6]. High-income countries, just 16% of the global population, account for 40% of all blood donations [1]. Still, shortages remain a concern even in these regions, e.g., the American Red Cross recently reported its lowest donor turnout in two decades [7]. WHO data highlights disparities in blood access across income levels: high-income countries report a median of 31.5 donations per 1,000 people, compared to 16.4 in upper-middle-income, 6.6 in lower-middle-income, and 5.0 in low-income countries [1]. While no official blood donation rate exists for Georgia, as an upper-middle-income country, its rate likely falls within that range [8].

Donors are typically classified into three groups: voluntary non-remunerated, family/replacement, and paid [9]. Voluntary donors give blood or components without payment, though they may receive compensation for travel or time [2]. Family/replacement donors give at the request of a patient or community member, while paid donors receive compensation or material benefits [2]. Even when not formally compensated, informal payments are sometimes involved. Regular, unpaid, voluntary donation remains the most effective strategy for ensuring a safe and sustainable blood supply [9].

According to WHO, voluntary, unpaid donations have increased in many low- and middle-income countries, with 79 nations now sourcing over 90% of their supply from such donors [1]. However, in 54 countries, the majority of blood still comes from family/replacement or paid donors [1]. In Georgia, approximately 60% of donations remain non-voluntary [3]. Efforts to shift toward full voluntary donation are ongoing, notably through the European Union Twinning Project [10]. This reform, aimed at ensuring a reliable supply of safe blood products, is being implemented gradually, with a full transition to unpaid voluntary donations expected by July 1, 2025 [10].

Globally, extensive literature has explored public knowledge and attitudes toward blood donation [11,12], identifying them as key determinants of donor behavior [13]. These are influenced by cultural norms, education, and access to information. Understanding the sociodemographic characteristics of potential donors is also essential for developing effective recruitment strategies [1]. International studies frequently cite lack of awareness and misconceptions about eligibility and the donation process as major barriers to voluntary donation [11,12].

To date, no published research has investigated public knowledge or attitudes toward blood donation in Georgia. This study is the first to investigate public knowledge and attitudes toward blood donation in Georgia. It aims to assess awareness of its importance and eligibility, identify misconceptions, and evaluate future donation intent, thereby informing national strategies for voluntary blood donation reform.

This research was previously presented as an abstract at the 57th Annual Meeting of the German Society for Transfusion Medicine and Immunohematology (DGTI) together with the 31st Annual Meeting of the German Society for Immunogenetics (DGI) on September 11, 2024.

## Materials and methods

Study design

This cross-sectional study examined the knowledge and attitudes of the Georgian population toward blood donation. Data collection was conducted from August to October 2023 using an online survey created in Google Forms. The survey link was distributed through non-randomized voluntary response sampling across various social media platforms. Participation was entirely voluntary, and no incentives were offered.

Sample size, inclusion and exclusion criteria

A minimum sample size of 385 participants was calculated using Raosoft's online sample size calculator, with a 95% confidence level, 5% margin of error, and a response distribution of 50%. Individuals aged between 18 and 60 years were eligible for inclusion. Participants were excluded if they were under 18 or over 60 years of age, did not provide informed consent, submitted incomplete or duplicate responses, or were unable to understand the survey language. All responses were complete, and no participants met the exclusion criteria.

Questionnaire development

The questionnaire was developed in collaboration with a transfusion specialist and five individuals affiliated with a blood donation center. It was produced in both English and Georgian and reviewed by a bilingual expert to ensure linguistic accuracy. A pilot test was conducted with 20 individuals (10 affiliated with blood donation and 10 not affiliated), and adjustments were made based on their feedback.

The final version (see Appendix) consisted of 17 questions: 14 multiple-choice and three checkbox-type items. These covered sociodemographic data, medical history (yes/no format), past donation behavior, reasons for not donating, future donation willingness, and awareness of blood group. Knowledge-related items assessed understanding of donation intervals, procedure duration, volume donated, and eligibility criteria. Participants were classified as knowledgeable if they answered at least seven out of 11 knowledge-based questions correctly. A positive attitude was defined by selecting "safe and important" when describing blood donation.

Statistical analysis

Categorical variables, including gender, residence, education level, and medical background, were reported as percentages. Age was presented as a mean with standard deviation (SD). Associations between knowledge or attitude and independent variables were assessed using Pearson’s chi-squared test. A 95% confidence interval (CI) and a p-value threshold of <0.05 were used to determine statistical significance. Analyses were conducted using GraphPad Prism version 9.5.0 and IBM SPSS Statistics version 23.0 (IBM Corp., Armonk, NY, USA).

Ethical considerations

The study protocol was reviewed and approved by the Biomedical Research Ethics Committee of Tbilisi State Medical University (approval #4-2023/105). All participants received information about the study’s purpose, the voluntary nature of their participation, and assurances regarding confidentiality and anonymity.

## Results

Sociodemographic characteristics

A total of 385 individuals participated in the study. Among them, 218 were female (56.6%) and 167 were male (43.3%) (Table 1). The mean age was 37.5 years (±11.9 SD), ranging from 18 to 60 years. Nearly half of the respondents resided in Tbilisi, while 17.9% lived in Batumi and 34.3% in other regions of Georgia. Regarding educational attainment, 41.6% had completed secondary education, 32.2% held a bachelor’s degree, 22.1% a master’s degree, and 4.2% a Ph.D. A total of 23.4% reported having a medical background, whereas 76.6% did not.

**Table 1:** Sociodemographic Characteristics of Respondents (N = 385)

Variable	Frequency (n)	Percentage (%)
Gender		
Female	218	56.60%
Male	167	43.30%
Age (years)		
18-25	70	18.20%
26-35	85	22.10%
36-45	96	24.90%
46-60	134	34.30%
Mean ± SD	37.5 ± 11.9	
Place of Residence		
Tbilisi	184	47.80%
Batumi	69	17.90%
Other regions	132	34.30%
Education Level		
Secondary education	160	41.60%
Bachelor’s degree	124	32.20%
Master’s degree	85	22.10%
Ph.D.	16	4.20%
Medical Background		
Yes	90	23.40%
No	295	76.60%

Knowledge of blood donation

Only 35% of participants demonstrated a satisfactory level of knowledge regarding blood donation. While 68.8% correctly identified the typical volume of blood collected during a donation, only 27.3% knew that the minimum interval between two donations is two months. Awareness of eligibility criteria was variable, with 62.9% and 63.4% correctly recognizing pregnancy and hepatitis A, respectively, as contraindications. However, only 12.5% knew that blood can be donated three months after receiving a piercing or tattoo.

Associations between knowledge levels and sociodemographic factors are summarized in Table 2. Statistically significant associations were found for gender (p < 0.0001), age (p < 0.05), place of residence (p < 0.0001), and educational level (p < 0.0001). Higher knowledge levels were observed among younger participants, residents of Tbilisi, and those with higher education. Although individuals with a medical background tended to have greater knowledge, the association was not statistically significant (p = 0.058).

**Table 2:** Association Between Knowledge of Blood Donation and Sociodemographic Variables χ² = Chi-square statistic; df = degrees of freedom; p < 0.05 considered significant. Pearson’s Chi-square test was used to assess associations.

Variable	Knowledge Association	Test Statistic	p-value
Gender	Significant	χ²(1)=44.461	< 0.0001
Age	Significant	χ²(3)=8.003	0.046
Residence	Significant	χ²(2)=36.214	< 0.0001
Education level	Significant	χ²(3)=47.975	< 0.0001
Medical background	Not significant	χ²(1)=3.297	0.058

Attitudes toward blood donation

A positive attitude toward blood donation was observed in only 38% of respondents (Table 3). Positive attitudes were significantly associated with female gender (p < 0.0001), younger age (p < 0.01), residence in Tbilisi (p < 0.0001), higher educational attainment (p < 0.0001), and a medical background (p < 0.05).

**Table 3:** Association Between Attitude Toward Blood Donation and Sociodemographic Variables χ² = Chi-square statistic; df = degrees of freedom; p < 0.05 considered significant. Pearson’s Chi-square test was used to assess associations.

Variable	Attitude Association	Test Statistic	p-value
Gender	Significant	χ²(1)=40.751	< 0.0001
Age	Significant	χ²(3)=11.361	0.01
Residence	Significant	χ²(2)=36.779	< 0.0001
Education level	Significant	χ²(3)=36.213914	< 0.0001
Medical background	Significant	χ²(1)=5.418	0.02

Willingness to donate blood in the future

A majority of respondents (83%) indicated a willingness to donate blood in the future. This willingness was significantly associated with being female (p < 0.0001), younger (p < 0.05), residing in Tbilisi (p < 0.0001), having higher education (p < 0.001), and having a medical background (p < 0.05) (Table 4). Additionally, higher knowledge levels (p < 0.05) and a positive attitude (p < 0.0001) were significantly correlated with willingness to donate.

**Table 4:** Association Between Willingness to Donate in the Future and Sociodemographic/Knowledge/Attitude Variables χ² = Chi-square statistic; df = degrees of freedom; p < 0.05 considered significant. Pearson’s Chi-square test was used to assess associations.

Variable	Willingness Association	Test Statistic	p-value
Gender	Significant	χ²(1)=34.005	< 0.0001
Age	Significant	χ²(3)=9.212	0.03
Residence	Significant	χ²(2)=31.085	< 0.0001
Education level	Significant	χ²(3)=17.447	0.001
Medical background	Significant	χ²(1)=5.634	0.01
Knowledge level	Significant	χ²(1)=8.515	0.002
Positive attitude	Significant	χ²(1)=28.998	< 0.0001

Reasons for avoiding blood donation

The most commonly reported barriers to blood donation were lack of opportunity (29.2%), anemia (15.6%), other health conditions (21.2%), hypotension (12.7%), and general reluctance (12.7%). Less frequently cited reasons included fear of needles (2.7%) and fear of infection (2.2%).

## Discussion

This cross-sectional study was conducted to evaluate knowledge, attitudes, and willingness to donate blood in the Georgian population. This was the first study in Georgia that attempted to address the serious issue of the shortage of blood supply [1,3]. Both global and local demand for blood is high, and shortages carry with them severe risks, particularly for those requiring emergency transfusion, such as trauma, obstetric hemorrhage, and patients with chronic conditions such as anemia [14]. The assessment of knowledge and attitudes of the population is required to implement effective public health interventions.

Our findings demonstrate a significant gap in knowledge regarding blood donation in the general population. Only 35% of the respondents possessed an acceptable level of knowledge, which is significantly lower than those reported in other countries, e.g., Iraq (66.7%) [15], Saudi Arabia (60.2%) [16], and Malaysia (97.1%) [17]. This variation highlights the need for specially directed education campaigns in Georgia, particularly given that awareness regarding basic donation criteria, such as eligibility periods following tattoos or piercings, was extremely low.

Sociodemographic analysis revealed that female respondents, younger age, residents of the capital city, and respondents with higher education were significantly more knowledgeable. These correlations suggest that urbanization, access to education, and possibly greater exposure to public health messages may result in greater awareness. Yet, knowledge in respondents with a medical background did not significantly differ from the general population (p = 0.058). This finding is alarming and surprising. This concurs with a similar study conducted among Portuguese and Spanish nursing students, which also revealed inadequate knowledge regarding blood donation procedures [18]. This indicates that even among the health profession, there are knowledge gaps. There might be a need to incorporate content regarding blood donation into medical and nursing school curricula so that healthcare professionals are better informed and prepared to act as ambassadors for blood donation.

In terms of attitudes, just 38.4% of participants had a positive attitude towards blood donation, which is significantly less than in most similar international studies [12,15,19,20]. It is possible that misconceptions, cultural issues, or limited exposure to donation campaigns are responsible for a less positive perception towards blood donation in Georgia. As with knowledge, attitude was significantly associated with gender, age, residence, education, and, in this case, having a medical background. The positive impact of these variables suggests that public opinion can be altered through focused interventions.

Reasons given for not donating blood also provide insight into obstacles that need to be overcome. The most common reason cited was the lack of opportunity to donate (29.2%), followed by medical reasons such as anemia (15.6%), hypotension (12.7%), and other medical reasons (21.2%). These findings are consistent with German, Ethiopian, and Tanzanian research [21-23], where logistical and medical barriers also inhibited blood donation. Psychological barriers such as fear of needles (2.7%) and infection (2.2%) were also cited, albeit less. Both practical and psychological barriers, by facilitating access to donor centers, making available comprehensible information about donor eligibility, and debunking myths, can be overcome in order to raise donation rates.

Despite the low levels of knowledge and relatively negative attitudes, the majority of respondents (83%) indicated that they would be willing to donate blood in the future. This is a highly encouraging finding and suggests that there is a responsive foundation upon which recruitment and awareness campaigns can build. Willingness to donate was also strongly associated with more knowledge and a positive attitude, suggesting a clear mechanism by which educational interventions could translate into behavior change. On the other hand, studies in Nigeria, Bangladesh, South Africa, and Tanzania have shown that even with acceptable levels of knowledge, intention to donate does not always follow [24]. This discrepancy provides an opportunity for Georgia to capitalize on the public's latent motivation by transforming intention into behavior through increased outreach and donation infrastructure.

This study has several limitations. The use of a non-random, voluntary sampling design may limit generalizability, as individuals who choose to participate may differ systematically from the broader population. Furthermore, the web-based administration of the survey excluded individuals without internet access or adequate digital literacy, introducing potential selection bias. The reliance on self-reported data also makes the findings susceptible to social desirability and recall biases. Additionally, the absence of an interviewer may have led to misinterpretation of certain questions, particularly among respondents with lower educational backgrounds. Future studies may benefit from larger, more diverse samples and offline data collection methods to enhance representativeness and reproducibility.

## Conclusions

Our study provides useful data on knowledge, attitude, and willingness to donate blood in the population of Georgia. The findings point us to areas of need for public health education and promotion of awareness, such as campaigns for explaining eligibility criteria and dispelling myths. Educational interventions should also be extended to medical professionals and students to strengthen their role as community educators and role models. Furthermore, elimination of logistic barriers, e.g., limited donation centers, and advertising the health and social benefits of blood donation may help translate high willingness into donor behavior.

## Appendices

Questionnaire

1.     Select your gender

o    Female

o    Male

2.     How old are you? (Short answer)

3.     Where do you live? (Please specify the city or village where you currently live)

4.     What is your current education status?

o    Basic education (up to 9th grade)

o    Secondary education

o    Bachelor’s degree

o    Master’s degree

o    Doctorate

o    Vocational education

5.     Do you have a medical education or connection to the medical field?

o    Yes

o    No

6.     Have you ever donated blood? If yes, how many times?

o    Never

o    1

o    2

o    3

o    4

o    More than 4

7.     If you have ever donated blood, what was the reason?

o    For a family member/friend/acquaintance

o    In exchange for money

o    Voluntarily

8.     If you have never donated blood, select all the reasons that apply to you: (Multiple choice)

o    Anemia - low hemoglobin

o    Other health problems

o    Low blood pressure

o    Fear of needles

o    Fear of contracting an infection

o    Never had the opportunity

o    Never wanted to

9.     Do you want to donate blood in the future?

o    Yes

o    No

10. Do you know your blood type?

Yes

No

11. Select the statements you agree with: (Multiple choice)

Blood donation is important

Blood donation is safe for the donor

Blood donation increases the risk of transmitting infectious diseases like hepatitis, syphilis, HIV

Blood donation increases the risk of developing chronic illnesses

12. Approximately how much blood is taken during one donation?

100-200 ml

200-400 ml

400-600 ml

13. On average, how long does the procedure take from arrival at the blood bank to completion of donation?

15 minutes

30 minutes

1 hour

1.5 hours

2 hours

14. Can a person with a tattoo donate blood?

Yes

No

15. Can a person with a piercing donate blood?

Yes

No

16. What is the minimum required interval between two donations?

2 weeks

1 month

2 months

3 months

6 months

17. In which of the following situations is blood donation NOT allowed? (Select all that apply)

During pregnancy

3 months after childbirth

During menstruation

During lactation

3 months after surgery

3 months after getting a tattoo

3 months after getting a piercing

1 year after having hepatitis A (Botkin’s disease)

3 months after visiting a dentist
